# Evolution of Fe-Rich Phases in Thermally Processed Aluminum 6061 Powders for AM Applications

**DOI:** 10.3390/ma15175853

**Published:** 2022-08-25

**Authors:** Kyle Tsaknopoulos, Caitlin Walde, Derek Tsaknopoulos, Danielle L. Cote

**Affiliations:** 1 Worcester Polytechnic Institute, Worcester, MA 01609, USA; 2 Solvus Global, LLC, Worcester, MA 01605, USA; 3 Mott Corporation, Farmington, CT 06032, USA

**Keywords:** powder, aluminum, additive manufacturing, cold spray, microstructure

## Abstract

Gas-atomized powders are frequently used in metal additive manufacturing (MAM) processes. During consolidation, certain properties and microstructural features of the feedstock can be retained. Such features include porosity, secondary phases, and oxides. Of particular importance to alloys such as Al 6061, secondary phases found in the feedstock powder can be directly related to those of the final consolidated form, especially for solid-state additive manufacturing. Al 6061 is a heat-treatable alloy that is commonly available in powder form. While heat treatments of 6061 have been widely studied in wrought form, little work has been performed to study the process in powders. This work investigates the evolution of the Fe-containing precipitates in gas-atomized Al 6061 powder through the use of scanning and transmission electron microscopy (SEM and TEM) and energy dispersive X-ray spectroscopy (EDS). The use of coupled EDS and thermodynamic modeling suggests that the as-atomized powders contain Al_13_Fe_4_ at the microstructure boundaries in addition to Mg_2_Si. After one hour of thermal treatment at 530 °C, it appears that the dissolution of Mg_2_Si and Al_13_Fe_4_ occurs concurrently with the formation of Al_15_Si_2_M_4_, as suggested by thermodynamic models.

## 1. Introduction

Many metal additive manufacturing (MAM) techniques utilize rapidly solidified, gas-atomized powders as feedstock. In MAM, it is important to understand the feedstock microstructure prior to consolidation. Depending on the MAM process, certain aspects of the feedstock powder can be retained after consolidation and can affect the microstructure and properties of the final consolidated part. For example, in liquid-state MAM processes, such as selective laser melting (SLM) and thermal spray, features such as the porosity and oxide shell can be retained [1,2,3]. Additionally, in solid-state MAM, the majority of the initial microstructural features—secondary phases, grain boundaries—are retained, though deformed to varying degrees. A greater understanding of the process inputs—feedstock powders—can lead to a greater understanding of the process outputs—the final consolidated part. This is especially useful when considering models for these MAM processes.

Aluminum alloys are often used in aerospace and automotive applications for their combination of light weight, strength, toughness, ductility, and corrosion resistance. Some of these conventionally made parts are being replaced by those made with MAM due to better optimization of strength and corrosion resistance [4]. 

Al 6061 is an Al-Mg-Si age-hardenable alloy. It is used primarily for its high strength, low density, and economic appeal. Its predominate secondary phase is β (Mg_2_Si), with precursors β″ (Mg_5_Si_6_) and β′ (Mg_9_Si_5_) offering the most strengthening contribution. A typical heat treatment for Al 6061 includes a solutionization step to homogenize the solute in the matrix followed by a quench to retain the supersaturated matrix and finished with an aging step to uniformly nucleate and grow β″ and β′ [5]. Additionally, as with most Al alloys, Al 6061 contains a variety of Fe-rich intermetallics. This is due to the low solubility of Fe in Al [6]. These phases are AlFeSi-based and are in the form of brittle needles or platelets. Fe can form various phases, depending on the presence of other alloying elements. These phases include Al_12_(FeMn)_3_Si, Al_8_Fe_2_Si, Al_12_Fe_3_Si, and Al_15_Fe_3_Si_2_, which form preferentially depending on the local composition [6,7]. They are the first phases to form from the liquid during solidification and because of their high melting temperature, they are difficult to dissolve during a homogenization or solutionization treatment [5,6]. 

In solid-state MAM, it has been shown that thermally treating the feedstock powder prior to consolidation can affect the properties of the consolidated part [8,9,10,11,12,13,14,15]. Considering this, it is possible to alter the feedstock powder using heat treatments to optimize the properties of the consolidated part. However, it has been shown that, in powder form, Al 6061 does not behave as its wrought counterpart [16]. For example, Mg_2_Si has unique morphologies in the powder form not seen in wrought Al 6061 [17]. Additionally, the small grain size of the powders, originating from their rapid solidification, decreases diffusion distance and therefore times, greatly accelerating diffusional processes. This becomes especially important to consider when optimizing thermal processing parameters for powders [18,19,20]. 

With the expectation that secondary phases in gas-atomized powders can vary from their wrought counterparts, this work characterizes the AlFeSi intermetallics found in gas-atomized Al 6061 powders. These intermetallics that are retained during solid-state MAM processing have been shown to be brittle in nature and can lead to crack initiation and propagation, leading to a decrease in ductility [21]. Given this, it is important to understand the effect of thermal treatments on these intermetallic phases. Both the as-atomized and thermally treated powder conditions are considered here. Characterization was performed using extensive scanning and transmission electron microscope (SEM and TEM) and energy dispersive spectroscopy (EDS) analysis.

## 2. Materials and Methods

Commercially available gas-atomized Al 6061 powder (Valimet, Inc., Stockton, CA, USA) was used in this study. The powder was mechanically sieved using laboratory test sieves compliant with ASTM E11 to aid in the repeatability of selecting similarly sized powder particles for analysis; the final powder had d_10_ of 32 μm, d_50_ of 41 μm, and d_90_ of 54 μm [22]. Direct current plasma emission spectroscopy and inert gas fusion were used to determine the elemental composition of the powders (Table 1) [23,24]. The composition complies with the ASTM B209 standard for Al 6061 alloys [25].

Simulations were performed using computational thermodynamic and kinetic modeling software (Thermo-Calc, Stockholm, Sweden) with the exact elemental composition of the sample to predict the secondary phases and their stability. Both equilibrium and Scheil solidification diagrams were created using the TCAL8 database. The Scheil solidification calculations can be used to predict the phases present in the as-atomized powder microstructure, as this calculation is applicable to rapidly solidified materials, such as the gas-atomized powder used in this study. The equilibrium calculations can be used to predict the phases present in the thermally treated microstructure, by analysis at the thermal treatment temperature of 530 °C. 

The powder was studied in two conditions, as-atomized and thermally treated. The thermally treated samples were treated at 530 °C for 1 h to create a homogenous microstructure. Treatment was performed in a differential scanning calorimeter (DSC) (TA Instruments, New Castle, DE, USA) with a heating rate of 50 °C/min and cooling rate of 120 °C/min in a nitrogen environment for approximately 50 mg of powder. This treatment profile was chosen based on the work of Walde et al. [16].

A Microtrac FlowSync (Microtrac Retsch GmbH, Haan/Duesseldorf, Germany) was used to measure the particle size distribution and morphology of the powder.

Loose powder samples were sprinkled over carbon tape onto a mounting stub for SEM analysis. One sample of each condition was mounted in epoxy and prepared for analysis via mechanical grinding and polishing with a final polish using 0.05 μm colloidal silica. Samples were then characterized using a tungsten-source SEM (Zeiss EVO MA10) at an accelerating voltage of 10 kV. Both secondary and backscatter detectors were used.

Two samples of each condition were also characterized using a probe-corrected TEM (Titan Themis with ChemiSTEM, ThermoFisher Scientific, Waltham, MA, USA) and energy dispersive X-Ray spectroscopy (EDS) (Super-X, ThermoFisher Scientific, Waltham, MA, USA) at an accelerating voltage of 300 kV. Samples were prepared for TEM analysis using a gallium-focused ion beam (FIB) (Helios 660 Nanolab, ThermoFisher Scientific, Waltham, MA, USA). Parallel-sided lamella were lifted-out from powder particle cross-sections and attached to a Mo omni-grid. Samples were then thinned to a thickness of less than 100 nm, using an ion beam of 5 kV as the final step to minimize surface stresses. 

The secondary phases were quantified by segmenting the SEM backscatter micrographs and the TEM high-angle annular dark-field (HAADF) images using image analysis software (Stream, Olympus Corporation, Tokyo, Japan). In the HAADF image, the Fe-rich phases contrast as lighter than the matrix while the Mg-rich phases contrast darker. For SEM, 10 micrographs were analyzed, and for TEM, 3–4 micrographs were analyzed per powder condition.

## 3. Results and Discussion

**Figure 1:**
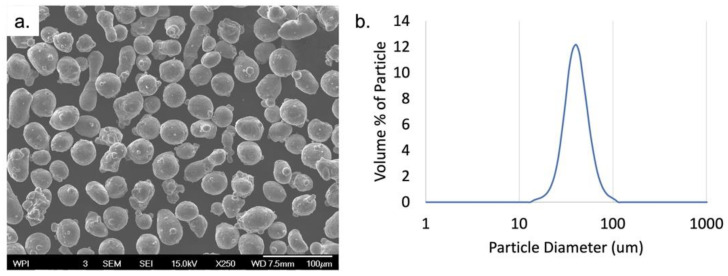
(**a**) BSE images of loose Al 6061 powder and (**b**) particle size distribution of Al 6061 powder.

In order to better understand the size and morphology of the powders used within this study, SEM micrographs were taken of the powder prior to cross-sectioning as seen in Figure 1a. This demonstrates the semi-spherical nature of the gas-atomized powders. The particle size distribution was also captured and shown in Figure 1b, confirming the size distribution was in line with the distribution described by the manufacturer. The average width-to-length ratio was also measured with a value of 0.77 ± 0.14, further demonstrating the semi-spherical nature of the powders.

**Figure 2:**
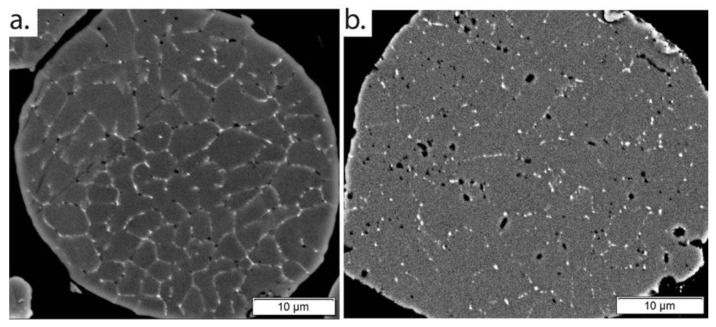
BSE images of cross sections of (**a**) the as-atomized powder and (**b**) the thermally treated powder.

Cross-sectional micrographs of the feedstock powder in (a) the as-atomized condition and (b) the thermally treated condition taken using a backscatter electron (BSE) detector are shown in Figure 2. In both, the light-contrasting regions correspond to the Fe-rich phases whereas the dark-contrasting regions are Mg_2_Si as determined by the reference [17]. In the as-atomized condition, Fe-rich phases decorate the granular boundaries completely, with small Mg_2_Si, primarily at the grain corners. This is typical of other gas-atomized Al 6061 powders that have been analyzed in literature [8,16,17,26]. In the thermally treated condition, the Fe-rich phases behave in two ways: those in the continuous phase structure along the boundary partially dissolve, while some discrete phases coarsen. Similarly, the Mg_2_Si phase also exhibits two different behaviors: some dissolve and disappear while others grow and stabilize. In both conditions, there is no nucleation observed in the bulk of the grain. However, it is widely acknowledged that the interaction volume of an SEM beam is insufficiently small to accurately identify phases of this size. Thus, TEM has been employed to further study microstructural evolution. 

**Figure 3:**
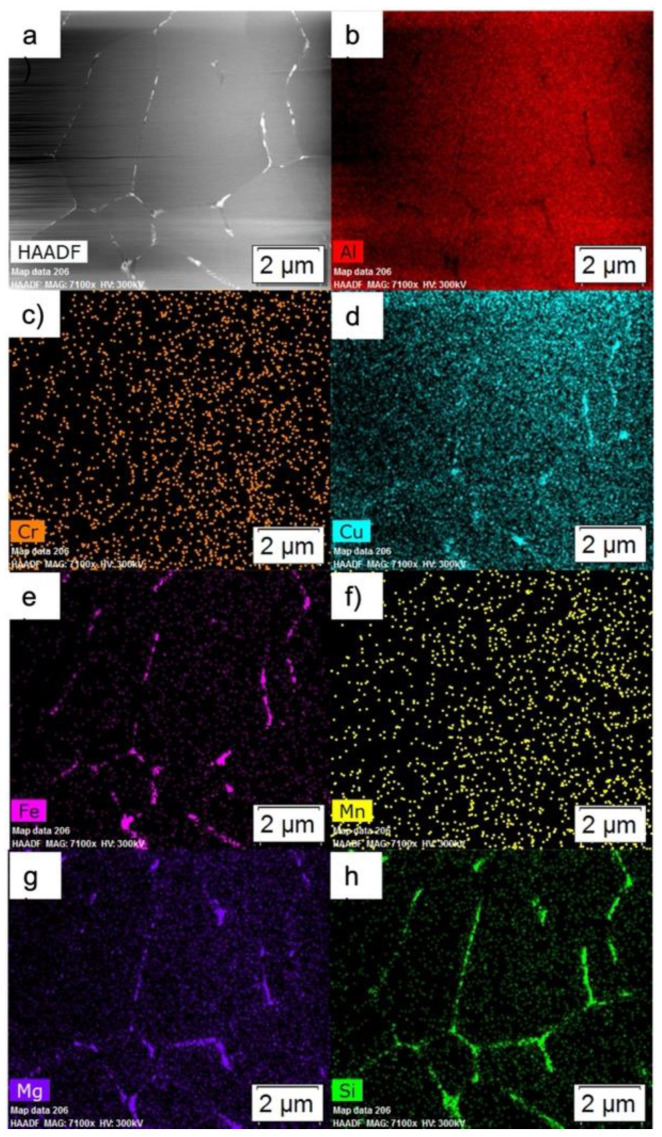
Overview images of the as-atomized powder: (**a**) HAADF, and EDS maps of (**b**) Al, (**c**) Cr, (**d**) Cu, (**e**) Fe, (**f**) Mn, (**g**) Mg, and (**h**) Si.

A TEM STEM HAADF image and overview EDS maps of the as-atomized condition are shown in Figure 3. Note that what appeared to be a continuous phase structure at the boundaries in SEM micrographs is comprised of discrete Fe-rich phases and small Mg_2_Si. The EDS here indicates that the Fe-rich phase is an Fe-Si-Al phase. Additionally, these micrographs confirm that there are no secondary phases in the bulk of the grains.

**Figure 4:**
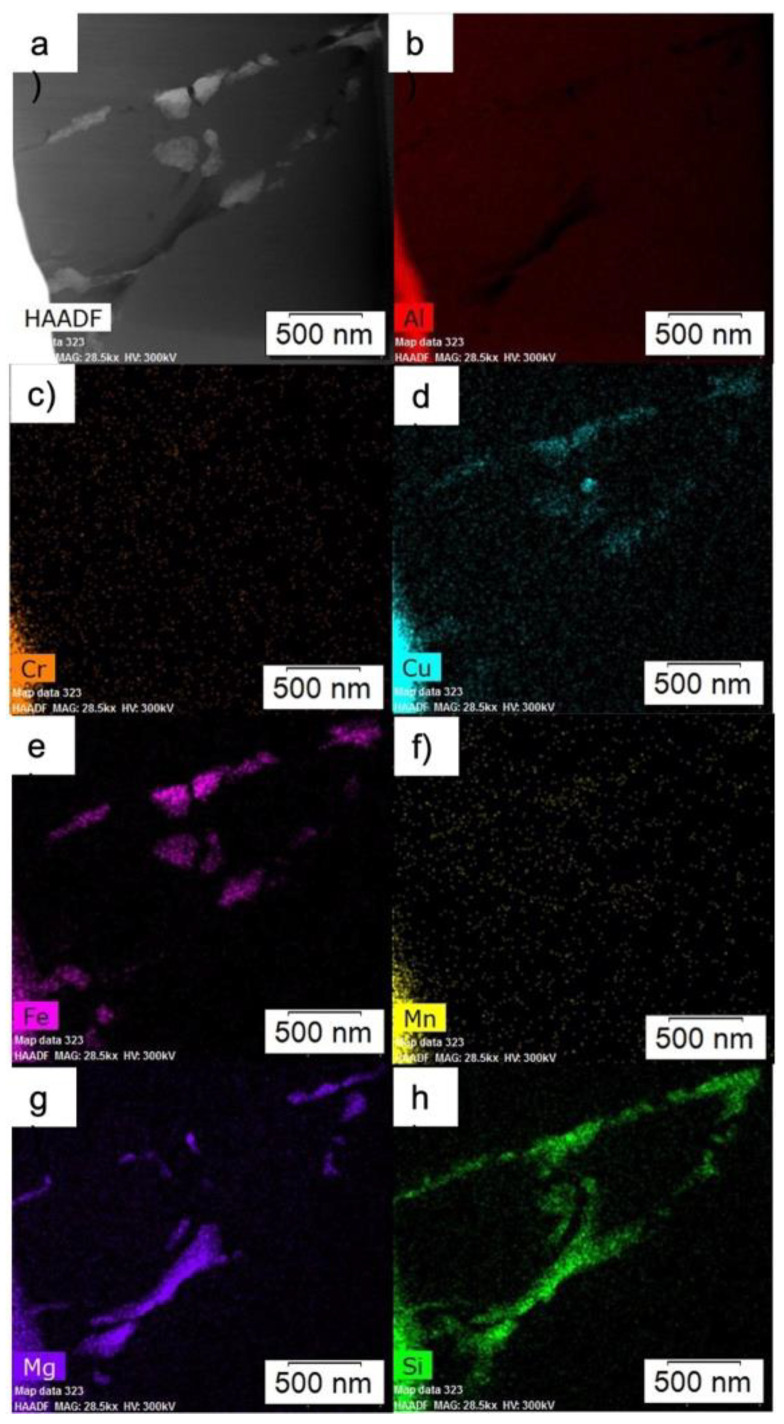
High magnification (**a**) HAADF, and EDS maps of (**b**) Al, (**c**) Cr, (**d**) Cu, (**e**) Fe, (**f**) Mn, (**g**) Mg, and (**h**) Si of a boundary in the as-atomized powder.

**Figure 5:**
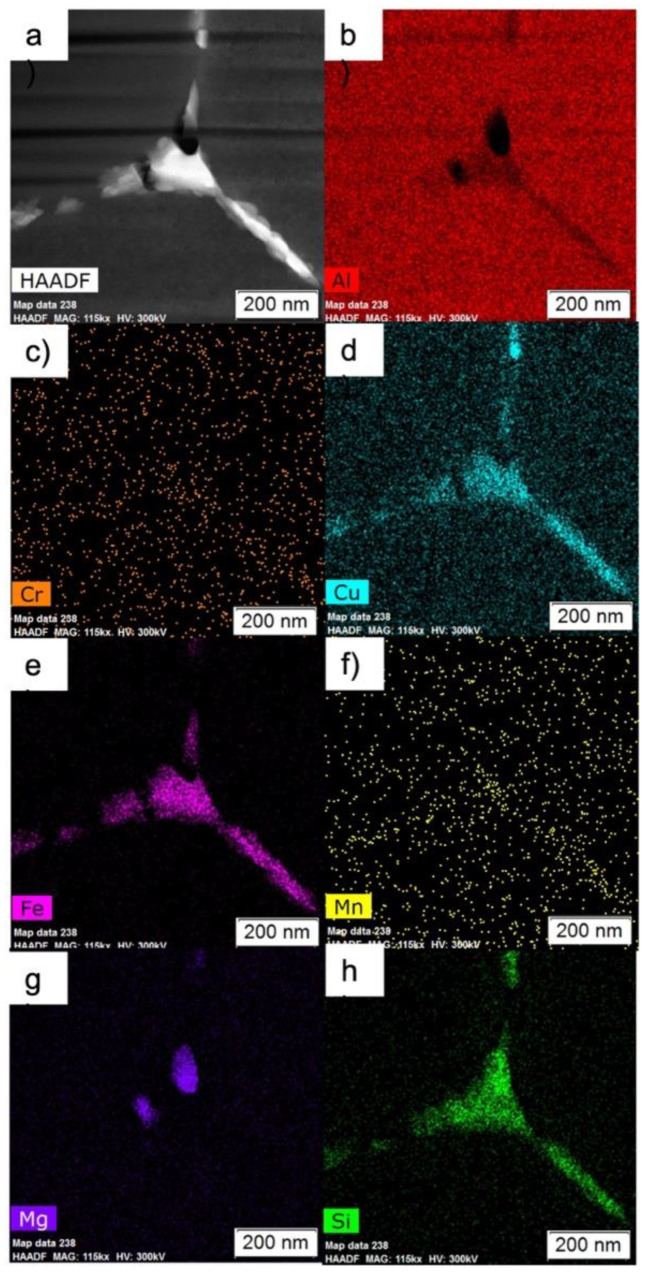
High magnification (**a**) HAADF, and EDS maps of (**b**) Al, (**c**) Cr, (**d**) Cu, (**e**) Fe, (**f**) Mn, (**g**) Mg, and (**h**) Si showing Mg_2_Si and Al_13_Fe_4_ at a triple point in the as-atomized powder.

High magnification of HAADF and EDS maps of a grain boundary in the as-atomized condition is shown in Figure 4. Note how the Fe-rich phases and Mg_2_Si are alternating on the boundaries, where the phases appear as stripes of alternating Fe-rich phases and Mg_2_Si. Figure 5 demonstrates a high magnification HAADF image of a triple point boundary in the as-atomized condition. Again, the Fe-rich phases and Mg_2_Si are alternating with one another. EDS here further indicates that the Fe-rich phase is an Fe-Si-Al phase. 

**Figure 6:**
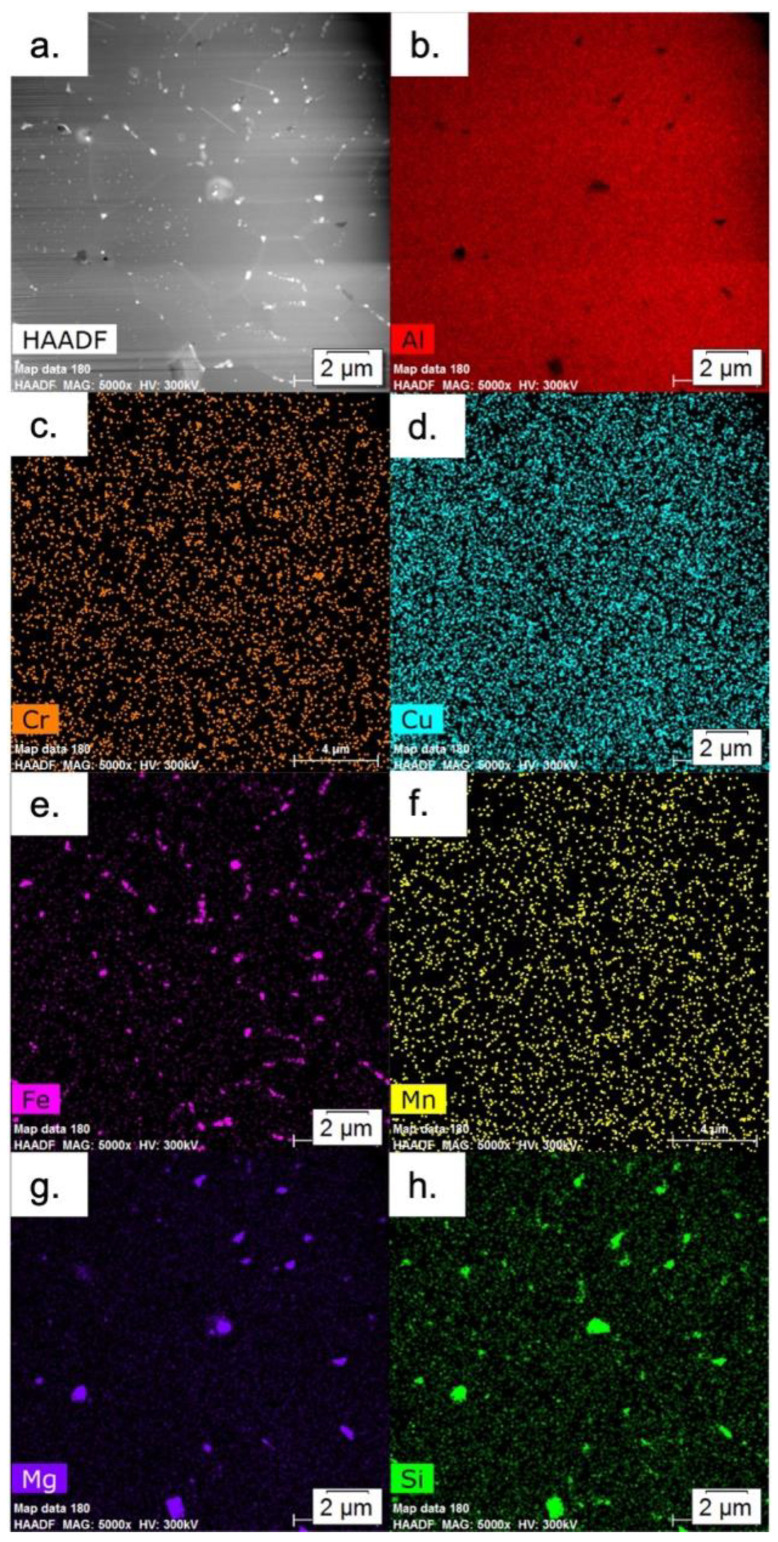
Overview images of the thermally treated powder: (**a**) HAADF, and EDS maps of (**b**) Al, (**c**) Cr, (**d**) Cu, (**e**) Fe, (**f**) Mn, (**g**) Mg, and (**h**) Si.

**Figure 7:**
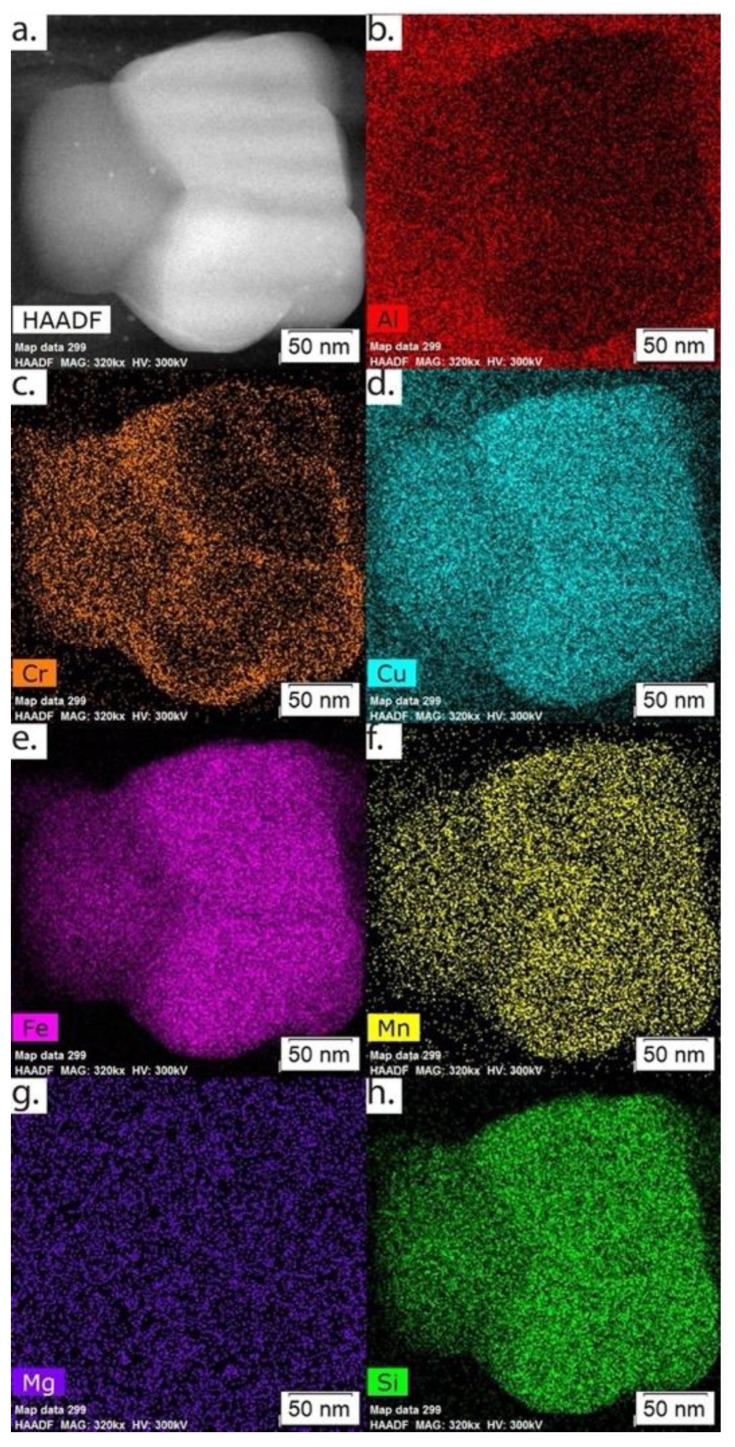
(**a**) HAADF, and EDS maps of (**b**) Al, (**c**) Cr, (**d**) Cu, (**e**) Fe, (**f**) Mn, (**g**) Mg, and (**h**) Si showing the morphology of Al_15_Si_2_M_4_ in the thermally treated powder.

A HAADF image and overview EDS maps of the powder microstructure in the thermally treated condition using TEM are displayed in Figure 6. Note that the Fe-rich phases and Mg_2_Si are no longer alternating and that the Mg_2_Si at the grain corners has substantially grown. Figure 7 displays high magnification HAADF and EDS maps of an Fe-rich phase, noting the incoherent boundaries, and the accumulation of Cr in the phase.

**Figure 8:**
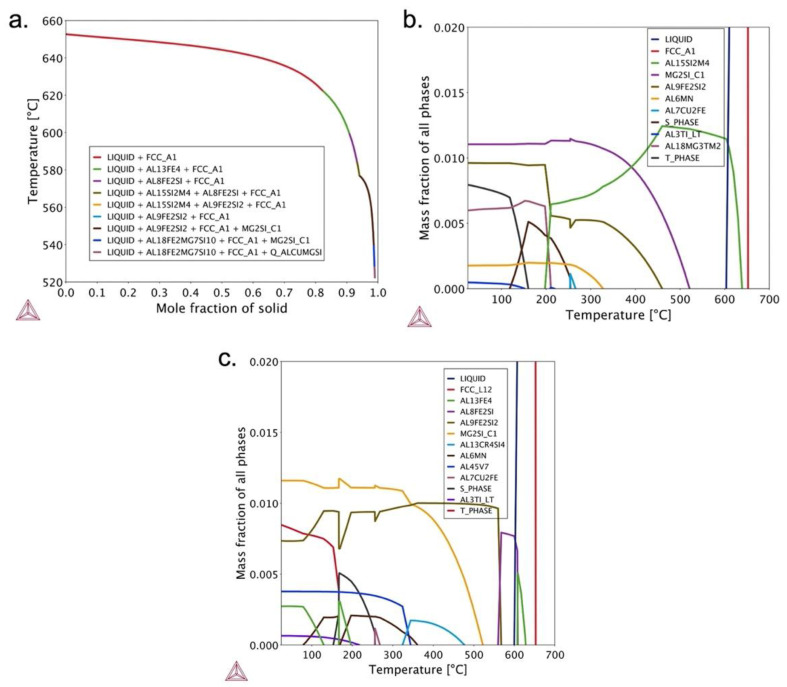
(**a**) Scheil solidification diagram and (**b**) equilibrium diagram for Al 6061 powder composition using ThermoCalc database TCAL8, (**c**) equilibrium diagram for Al 6061 powder composition using ThermoCalc database TCAL5.

The Scheil solidification and equilibrium diagrams using the exact powder composition are found in Figure 8a and Figure 8b, respectively, indicating the predicted secondary phases for the as-atomized and thermally treated powders, respectively. Table 2 demonstrates the volume percentage of the predicted secondary phases for the as-atomized and thermally treated powder conditions. The main phases predicted for the as-atomized condition are the Al matrix, Mg_2_Si, and Al_13_Fe_4_, while the predicted phases for the thermally treated condition are the Al matrix and Al_15_Si_2_M_4_. These predictions suggest that the Mg_2_Si should fully dissolve at the chosen treatment temperature, while the Al_13_Fe_4_ should transform into Al_15_Si_2_M_4_ once the system has reached equilibrium.

**Figure 9:**
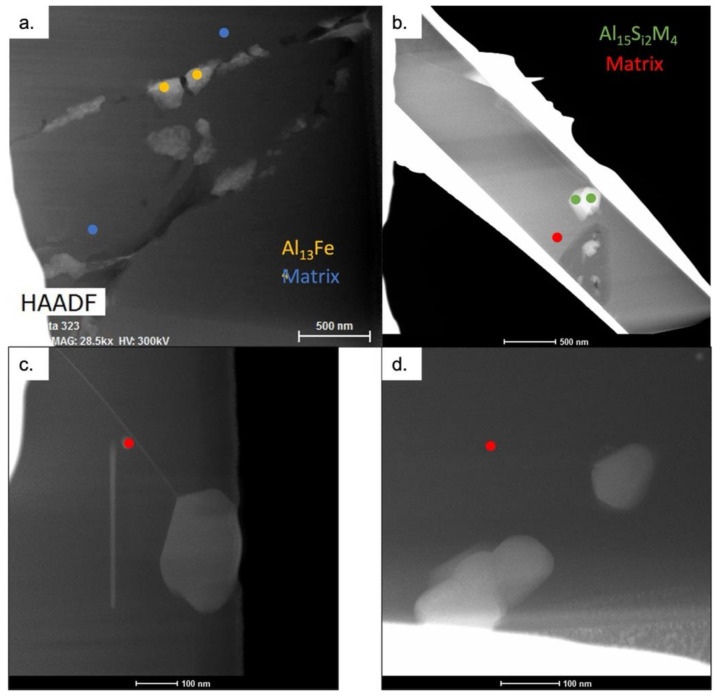
TEM micrographs depicting the locations of measurements taken for calculation of phase compositions in Table 3 for the (**a**) as-atomized, and (**b**–**d**) thermally treated at 530 °C microstructures.

The compositions of the Al-Fe-Si phases and Al matrix phases as quantified by TEM point EDS analysis and compared to the main phase compositions predicted by ThermoCalc for both the as-atomized and thermally treated conditions are shown in Table 3. The locations of the phase composition values measured within the calculations in Table 3 are depicted in the TEM micrographs found in Figure 9. The combination of the predicted volume percentages of phases found in Table 2 and the compositions found in Table 3 suggest that the Al-Fe-Si phase present in the as-atomized microstructure is Al_13_Fe_4_. While the predicted phase volume fractions in Table 2 for the as-atomized powder suggest the presence of several Al-Fe-Si phases, the TEM EDS suggests the presence of just one phase composition, containing about 5 wt % Cu. Al_13_Fe_4_ is the only one of the phases predicted by Thermo-Calc that allows for the substitution of Cu into the phase as shown by the exact phase stoichiometry from ThermoCalc in Table 4, suggesting further that the phase found experimentally is Al_13_Fe_4_. Given that, it would be expected that all of the Fe and Si would be used up for the formation of additional Al_13_Fe_4_, which could cause an increase in the Al_13_Fe_4_ volume fraction when compared to the amount in Table 2. It should be noted that the measured values from the TEM EDS for the phases did not perfectly match the predicted compositions; this is likely due to spatial resolution limitations of EDS even at the TEM scale. There is still the possibility that the measurements picked up signals from the Al matrix around or potentially behind the secondary phases. Despite this, the general compositional trends were consistent with the predicted results.

An increased level of Mg and Si in the Al_13_Fe_4_ EDS quantification when compared to the predicted results was also observed, which can be explained by the proximity of the Mg_2_Si phases to the Al_13_Fe_4_ at the grain boundaries. However, according to the measured results in Table 3, Cu was measured at higher percentages in the Al_13_Fe_4_ than was predicted. This measured phase should be sufficiently large to obtain reasonably accurate measurements. Cu seems to have an affinity for Al_13_Fe_4_ more so than predicted, as seen by the increased amount of Cu in the Al_13_Fe_4_ when compared to the predicted, and the decrease in Cu in the Al matrix when compared to the predicted values, suggesting a discrepancy between the Thermo-Calc database and the measured phase composition. This could be due to the differences between the wrought and cast data used in database creation when compared to the actual phenomena present in rapidly solidified gas-atomized powder and could be expected when utilizing models.

In the thermally treated condition, Table 3 shows good agreement between the measured composition values for the Al-Fe-Si phase and the predicted values for Al_15_Si_2_M_4_. It is hypothesized here that the Al_13_Fe_4_ present in the as-atomized conditions transforms into Al_15_Si_2_M_4_ upon thermal treatment. A distinct composition change in both the predicted and measured is seen between the Al-Fe-Si phases from the as-atomized to thermally treated condition. The predicted composition change from Al_13_Fe_4_ to Al_15_Si_2_M_4_ demonstrated a large increase in Si, Cr, and Mn, a slight increase in Al, and a decrease in Fe, Zn, and Cu. The measured phase compositions demonstrated corresponding increases in Cr, Mn, and Al, and decreases in Cu and Zn corroborating the identity of the Al_15_Si_2_M_4_ phase. A large increase in Si between the two conditions, as predicted by Thermo-Calc, was expected but instead a decrease in the Si content was seen. This is likely due to the remaining, unpredicted Mg2Si present in the thermally treated microstructure, depleting the additional Si solute available. 

Elevated amounts of Cu and Mg were seen in the measured composition of the Al_15_Si_2_M_4_ phase when compared to the predicted values. This is likely due to interaction from the surrounding matrix, as there were similar amounts of these elements present in the measured matrix values in Table 3. According to the phase stoichiometry in Table 4, these elements are also not able to be present within the phase matrix.

**Figure 10:**
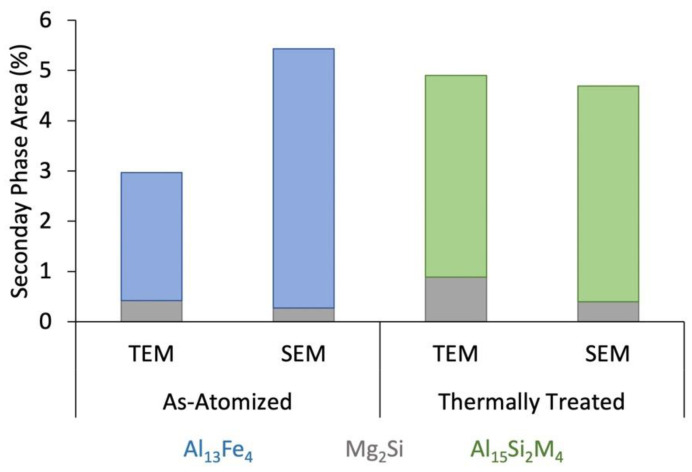
Secondary phase area percentages were determined for the as-atomized and thermally treated conditions via both SEM (points) and TEM (bars).

In addition to comparing the composition of predicted versus measured phases, area fractions of the identified phases were calculated from the micrographs to compare to predicted volume fractions. Image thresholding was used to quantify the amounts of the secondary phases present in the microstructure. Figure 10 shows the quantification results for both the SEM and TEM. For the as-atomized condition quantified via SEM, it was determined that there was 0.27 ± 0.27% (area) Mg_2_Si and 5.16 ± 2.25% (area) Al_13_Fe_4_, while via TEM it was determined that there was 0.4 ± 0.8% (area) Mg_2_Si and 2.5 ± 1.23% (area) Al_13_Fe_4_. The area percentages measured for the Mg_2_Si were consistent between the SEM and TEM quantifications. However, for the Fe-rich phase, Al_13_Fe_4_, the SEM measured twice as much as the TEM. This elevation is likely due to the larger interaction volume in the SEM, which makes phases under the surface fluoresce. Additionally, due to the small size of the TEM samples, it is expected that the exact quantifications may only be approximate, though the trends are likely still significant. 

For the thermally treated condition as measured via SEM, it was determined that there was 0.4 ± 0.3% (area) Mg_2_Si and 4.29 ± 1.22% (area) Al_15_Si_2_M_4_, while via TEM it was determined that there was 0.9 ± 0.6% (area) Mg_2_Si and 4.0 ± 0.6% (area) Al_15_Si_2_M_4_. Similar to the as-received condition, the area fractions measured for the Mg_2_Si were consistent between the SEM and TEM quantifications. Unlike the as-atomized condition, the amount of the Fe-rich phase, Al_15_Si_2_M_4_, measured in the SEM was consistent with that measured in the TEM. Since the Al_15_Si_2_M_4_ precipitates are larger and more coarsened after thermal treatment than the Al_13_Fe_4_ precipitates were in the as-atomized condition, they were easier to isolate for more accurate measurements. Again, due to a small TEM sample size, it is expected that the exact quantifications may only be approximations for the actual amount of phases present. 

When comparing the as-atomized to thermally treated conditions in Figure 10, it appears that the area fraction of Mg_2_Si, as determined via both SEM and TEM, increases slightly in the thermally treated condition. This is inconsistent with what is predicted, given that the Mg_2_Si should dissolve at the treatment temperature of 530 °C. When comparing the phase fractions between the experimental values from Figure 6 and the predicted values found in Table 2, the as-atomized condition had ~0.4% Mg_2_Si in the experimental values, compared to 0.67% Mg_2_Si in the predicted values, which shows fairly good agreement when considering the error inherent to the measurements. The thermally treated condition had no Mg2Si predicted compared to the 0.9% Mg_2_Si that was measured. When the micrographs were evaluated qualitatively, it can be seen that there were many small Mg_2_Si precipitates in the as-atomized condition as opposed to the few, larger Mg_2_Si precipitates in the thermally treated condition; this could lead the overall area fractions to be similar, but with different size distributions. This is consistent with other studies of gas-atomized Al 6061 powders [16,17]. The lack of complete dissolution of Mg_2_Si could be due to differences in local equilibrium due to local chemistry differences when compared to the alloy composition, or the thermal treatment time could be increased to promote further dissolution. 

When comparing the phase fraction of the AlFeSi phases in the as-atomized and thermally treated conditions in Figure 10, there was an increase in the phase fraction according to TEM, and a decrease according to SEM. Due to the small sample size from TEM, the true volume fraction may have been skewed. In the SEM, there were very large error bars, suggesting that there could be an overlap in the potential “true” volume fraction value for these phases. When comparing these values to the predicted volume fractions of the phases the as-atomized microstructure had ~4% measured Al_13_Fe_4_, while 0.5% of all AlFeSi phases were predicted. The thermally treated condition microstructure had ~4% measured Al_15_Si_2_M_4_, while 0.88% was predicted. This is a large discrepancy which is likely partly caused by the fluorescence in microscopy imaging, particularly of light contrasting Fe phases, and inherent difficulties in thresholding image analysis. Given this alloy composition, since the Mg_2_Si volume fraction is close to that of the predicted value, it is thermodynamically implausible that the fraction of any of the AlFeSi phases could be as high as the measured values since there is not enough solute available to allow for it. 

It is important to remember that the type of sample considered here is a powder that has been rapidly solidified. Given the finer microstructural features in powders, compared to their wrought counterparts, the shorter diffusion distances and metastable conditions have a great effect on the phase type, morphology, and chemistry seen in the powders [27]. This is consistent with other microstructural evaluations of rapidly solidified powders [16,17]. The phases typically seen in bulk Al 6061 are Al_12_(FeMn)_3_Si, Al_8_Fe_2_Si, Al_12_Fe_3_Si, and Al_15_Fe_3_Si_2_ [6,7], which are different from those seen in the powder used in this study. Based on equilibrium conditions, Al_9_Fe_2_Si_2_ would be present in abundance at room temperature. However, these powders were rapidly solidified, undergoing nonequilibrium conditions. Based on Scheil solidification (nonequilibrium conditions) predictions, Al_13_Fe_4_ was the most abundant phase formed during solidification.

An interesting observation can also be made here about the improvements in thermodynamic databases over time. Tsaknopoulos et al. performed work in a similar system, addressing the Mg_2_Si evolution [17]. Figure 2 in their work shows the thermodynamic stability of phases in this system. As indicated in Figure 2, Al_13_Fe_4_ was less stable than Al_9_Fe_2_Si_2_ at 530 °C, the treatment temperature used here. Therefore, it was hypothesized that, upon heating, the Al_13_Fe_4_ would dissolve, and the Al_9_Fe_2_Si_2_ would readily form [17]. Those diagrams were created using the ThermoCalc database TCAL5 while the diagrams in Figure 7b and Figure 8a presented here were created using an updated database, TCAL8. With the updated database, there is a significant change to the equilibrium diagram when compared to one created using the older database, as seen in Figure 8c. As mentioned, according to the diagram in Figure 8c, the most abundant phase at the thermal treatment temperature should be Al_9_Fe_2_Si_2_, while in Figure 8b with the updated data, the most abundant phase at that temperature is the Al_15_Si_2_M_4_. This is a very significant update to the database, given that this change allows for a much better understanding of the experimental data compiled in this study. If the older database were used, suggesting the presence of the Al_9_Fe_2_Si_2_ phase, when comparing the measured composition of the phases in the thermally treated powders to the predicted, it would be evident that there is an excess of Cr in the phase that, according to the stoichiometry from Table 4, is not possible, leading to inconclusive results. With the updated database, the identity of the AlFeSi phases in the thermally treated powder matches the composition of the Al_15_Si_2_M_4_ phase more accurately, accounting for the increased Cr content due to the fact that the newer database update introduced the presence of Cr and Mo into the Al_15_Si_2_M_4_ phase. This highlights the importance of ongoing updates to thermodynamic databases to allow for more complete analysis in alloy systems. While these are incredibly powerful tools, some experimental characterization of novel material systems is still necessary for the constant growth of these predictive models. 

## 4. Conclusions

Through the use of thermodynamic models, this work analyses the effect of thermal treatment on the AlFeSi intermetallics present in gas-atomized Al 6061 powders by employing SEM, TEM, and EDS analysis. The results suggested that Al_13_Fe_4_ is present in the as-atomized condition and transforms to Al_15_Si_2_M_4_ after a treatment of 1 h at 530 °C. Since parts made via MAM techniques can retain microstructural features of the feedstock powder in the consolidated part, it is beneficial to understand the microstructure of the feedstock powder prior to consolidation. A complete understanding of the secondary phases present in these powders can help aid in future prediction of powder and final cold spray consolidation properties.

This work highlights the importance of using a combination of advanced microscopy guided by thermodynamic modeling in order to drastically decrease the amount of time and number of samples necessary for microstructural analysis through SEM and TEM. This can allow for the rapid iteration of material conditions as well as reduce the number of experiments necessary to optimize thermal treatment of materials. These models can be used to supplement experiments, with a much smaller amount of experimental work necessary to validate the results, which can also aid in further updates to thermodynamic databases. 

## Figures and Tables

**Table 1:** Elemental composition of studied powder and ASTM Standard.

Element	Studied Powder	ASTM B209
	wt. %	wt. %
Magnesium	0.950	0.80–1.20
Silicon	0.490	0.40–0.80
Iron	0.270	<0.70
Copper	0.250	0.15–0.40
Oxygen	0.100	---
Chromium	0.087	0.04–0.35
Zinc	0.035	<0.25
Manganese	0.034	<0.15
Titanium	0.024	<0.15
Other	---	<0.15
Aluminum	Remainder	Remainder

**Table 2:** Volume percentage of secondary phases predicted by ThermoCalc for the as-atomized and thermally treated powder conditions.

Phase Name	As-Atomized	Thermally Treated 530 °C
Al_15_Si_2_M_4_	0.011	0.876
Al Matrix	98.798	99.124
Mg_2_Si	0.663	
Al_13_Fe4	0.2558	
Al_8_Fe_2_Si	0.1462	
Al_9_Fe_2_Si_2_	0.125	

**Table 3:** Phase compositions (in wt. %) as calculated by TEM point EDS and as predicted by ThermoCalc for the as-atomized and thermally treated conditions.

	**As-Atomized**		**Thermally Treated 530 °C**	
	**Al_13_Fe_4_**		**Al_15_Si_2_M_4_**	
Element	Predicted	Measured	Predicted	Measured
Al	57.28	68.05 ± 0.39	61.1	72.82 ± 4.99
Cu	0.022	4.87 ± 0.32	0	1.70 ± 0.14
Fe	38.8	13.02 ± 0.22	21.96	13.79 ± 2.09
Mg	0	1.12 ± 0.09	0	0.38 ± 0.12
Mn	0.00038	0.21 ± 0.02	1	1.32 ± 0.42
Si	3.75	12.56 ± 0.56	9.02	8.53 ± 1.91
Cr	0	0.13 ± 0.00	6.92	1.44 ± 0.55
Zn	0.15	0.04 ± 0.01	0	0.01 ± 0.00
Ti	0	0.01 ± 0.01	0	0.01 ± 0.01
	**As-Atomized**		**Thermally Treated 530 °C**	
	**Al Matrix**		**Al Matrix**	
Element	Predicted	Measured	Predicted	Measured
Al	96.07	97.35 ± 0.21	98.312	96.99 ± 0.77
Cu	1.93	0.70 ± 0.11	0.253	1.26 ± 0.53
Fe	0.00077	0.05 ± 0.00	0.003	0.11 ± 0.06
Mg	0.47	1.56 ± 0.38	0.962	0.77 ± 0.32
Mn	0.018	0.01 ± 0.00	0.022	0.00 ± 0.00
Si	1.28	0.29 ± 0.09	0.385	0.84 ± 0.64
Cr	0	0.00 ± 0.00	0.003	0.01 ± 0.01
Zn	0.23	0.02 ± 0.02	0.035	0.00 ± 0.00
Ti	0	0.00 ± 0.00	0.022	0.02 ± 0.02

**Table 4:** Secondary phase stoichiometry from Thermo-Calc database.

Phase Name	Stoichiometry
Al_13_Fe_4_	(Al,Cu)_0.63_(Zn,Fe,Mn)_0.23_(Al,Si,Zn)_0.14_
Al_15_Si_2_M_4_	Al_16_(Si,Al)_2_(Mn,Fe,Cr)_4_Si_1_
Al_8_Fe_2_Si	Al_0.66_(Fe,Mn)_0.19_Si_0.05_(Al,Si)_0.1_
Al_9_Fe_2_Si_2_	Al_0.6_(Fe,Mn)_0.15_Si_0.1_(Al,Si)_0.15_

## Data Availability

Not applicable.
